# Who's afraid of epigenetics? Habits, instincts, and Charles Darwin’s evolutionary theory

**DOI:** 10.1007/s40656-021-00376-9

**Published:** 2021-02-10

**Authors:** Mariagrazia Portera, Mauro Mandrioli

**Affiliations:** 1 Dipartimento di Lettere e Filosofia, University of Florence, Firenze, Italy; 2 Dipartimento di Scienze della Vita, University of Modena e Reggio Emilia, Modena, Italy

**Keywords:** Habit, Instinct, Epigenetic mechanism, Evolution, Transmission, Heredity, Transformism, Lamarck, Research methodology

## Abstract

Our paper aims at bringing to the fore the crucial role that habits play in Charles Darwin’s theory of evolution by means of natural selection. We have organized the paper in two steps: first, we analyse value and functions of the concept of habit in Darwin's early works, notably in his *Notebooks*, and compare these views to his mature understanding of the concept in the *Origin of Species* and later works; second, we discuss Darwin’s ideas on habits in the light of today’s theories of epigenetic inheritance, which describe the way in which the functioning and expression of genes is modified by the environment, and how these modifications are transmitted over generations. We argue that Darwin’s lasting and multifaceted interest in the notion of habit, throughout his intellectual life, is both conceptually and methodologically relevant. From a conceptual point of view, intriguing similarities can be found between Darwin’s (early) conception of habit and contemporary views on epigenetic inheritance. From a methodological point of view, we suggest that Darwin’s plastic approach to habits, from his early writings up to the mature works, can provide today’s evolutionary scientists with a viable methodological model to address the challenging task of extending and expanding evolutionary theory, with particular reference to the integration of epigenetic mechanisms into existing models of evolutionary change. Over his entire life Darwin has modified and reassessed his views on habits as many times as required by evidence: his work on this notion may represent the paradigm of a *habit* of good scientific research methodology.

## Introduction

The last few years have witnessed an impressive resurgence of interest in the notion of “habit” across a wide range of contemporary fields of inquiry: philosophers turn to the concept to investigate its significance to the historical development of Western thought (Carlisle 2014, 2018; Sparrow and Hutchinson 2013); cognitive scientists and neuroscientists investigate the role it has played in structuring our idea of the functioning of the human mind (Nöe 2009; Caruana and Testa 2020) and its neural and psychological underpinnings (Graybiel 2008); anthropologists, political scientists and sociologists have found in habits one of their key notions (Latour 2013; Pedwell 2017). Our paper fits into this line of inquiry: it aims at bringing to the fore the pivotal role that habits have played in the historical development of modern biology, with specific reference to Charles Darwin’s theory of evolution by means of natural selection, and the role this concept might still play in contemporary evolutionary biology.

As Carlisle (2018) points out, “habit”—a word coming, etymologically, from the Latin “habitus” (“habeo”, to have), which is, in turn, a calque of the Greek “hèxis” (from “èchein”, to have, “to hold a form through time”)—is a genuinely interdisciplinary concept, widely used in botany, mineralogy, zoology and of course anthropology and the human and social sciences. As she puts it, “mineralogists refer to the habits of crystals; botanists to the habits of plants; of course, animals, including humans, have habits—and in each case “habit” means a shape or pattern of growth […] Habits are the “way” in which […] an all-encompassing unity, expresses or manifests itself in diverse forms of life (Carlisle 2018, p. 105).

We contend that Charles Darwin’s theory of evolution—which investigates how in nature “from so simple a beginning endless forms most beautiful and most wonderful have been, and are being, evolved” (Darwin 1859, p. 490)—is uniquely positioned to address this all-encompassing unity of the diverse forms of life, so vividly witnessed, as Carlisle argues, by the concept of habit. In the theory of the great Victorian habits appear to be indeed crucial factors, not only historically but also conceptually and methodologically.

From an historical perspective, the high frequency of the concept, which recurs uninterruptedly from Darwin’s early *Notebooks* up to the last publications, is indicative of how much Darwin’s theory owes to the intellectual *milieu* of his time; as a matter of fact, “habit” was one of the most widely debated notions in the first half of the nineteenth century in England and Europe, in philosophy and biology. On the other hand, from a conceptual perspective, the *specific* ways in which Darwin understands and makes use of the notion, and the various meanings he attributes to it throughout his *opus*, prove how astonishingly *ahead of his time* he also was, at least under certain respects. As we will show, intriguing similarities can be found between Darwin’s early conception of habit and today’s theories of epigenetic inheritance. But what we find most interesting is, from a methodological point of view, the lesson we can globally take from Darwin’s discussion of habits: a lesson, we will argue, of flexibility, plasticity and pragmatism in the production of scientific knowledge.

Habits, then, are definitely at the heart of Darwin’s theory. Despite this cruciality, there has been so far only little research on their role and relevance to evolution (Richards 1981, 1987, 1992). Our paper aims to fill in this gap in current research, thus contributing with a new chapter both to the history and theory of Darwinism and to the current international and inter-disciplinary debate on the notion of “habit”.

In order to develop our argument, we have divided it into eight sections: in Sects. 2 and 3, we show that *habits*, together with animal *instincts*, count for the young Darwin as the *main* agents of evolution and the most fundamental mechanisms of species transformation. Influenced by the philosophical *milieu* of his time (Wright 2011, 2017) and in particular by Scottish transformism, Darwin uses the term “habit” to refer to recurrent patterns of behaviour, skills and practices acquired by an individual over the course of his lifetime, and performed, at least at the beginning, with a certain degree of voluntariness and consciousness.

Of particular relevance is the transition from this evolutionary view focused on voluntary habits (and on instincts) in Darwin’s early writings to an evolutionary view focused on the power of natural selection in the mature works (starting, paradigmatically, with the first edition of the *Origin of species*), in which the role of voluntary habits is significantly downsized. This decisive transition is analytically discussed in Sects. 4, 5 and 6, also with references to Darwin’s use of the term “habit” in his botanic investigations, which represent, as well known, a major part of his scientific endeavour.

In Sect. 7, we introduce today's models of epigenetic inheritance (which describe the way in which the functioning and expression of genes is modified by the environment, and how these modifications are transmitted over generations) and ask (Sect. 8) to what extent Darwin’s struggles with the notion of habit, moving from his *Notebooks* to the *Origin of Species*, can shed some new light on the challenges that today’s scientists face in integrating epigenetics into the current Darwinian synthesis.

Of course, Darwin did not know anything about genetics, not to speak of epigenetics, nor was he aware of the meticulous experimentations carried out by Gregor Mendel on pea plants in the second half of the nineteenth century. However, as Avital and Jablonka wrote (Avital and Jablonka 2000, p. 30) “it is to Darwin […] that we should turn again […], to his belief that individual and social habits drive much of the evolution of behaviour” in order to properly understand what epigenetics is and what is today at stake with it. What is the relationship between habits, habitual processes and epigenetics processes? What role do habitual processes play in heredity and, more in general, in evolution? What might Darwin’s “tormented” treatment of the notion of habit teach us about how theories can be expanded and extended without losing their conceptual core and overall coherence?

As mentioned, we think that the greatest lesson we may take from Darwin’s discussion of habits is perhaps a methodological one. Scholars have notoriously emphasised Darwin’s pluralist and pragmatic approach to the process of production of scientific knowledge (see Gruber and Barrett 1974; Gruber 1985). Over his entire intellectual life, the Victorian has always understood concepts more as flexible tools of discovery than as dogmas and has given more importance to flexibility and growth of science than to strict adherence to the theory. This is paradigmatically the case with Darwin’s treatment of the concept of habit. In the transition from the early *Notebooks* to his last publications, Darwin changes his mind about habits and amends his views as many times as required by evidence; in a multi-faceted and always-developing process of adjustment, he ends up anticipating views that are currently under debate among contemporary epigeneticists. Evolutionary scholars currently involved in the challenging task of expanding Darwin’s theory, in particular those interested in integrating into it the relatively new ideas about epigenetic inheritance, may find in Darwin’s flexible *habit* of science a useful methodological paradigm (Huneman 2014).

## The origin of habits

Francis Huxley, a grand-grandson of Thomas Henry Huxley, the English biologist who earned the nickname of "Darwin's bulldog" for his fiery advocacy of Charles Darwin's theory of evolution, wrote in 1959:

A structure to [Darwin] meant a habit, and a habit implied not only an internal need but outer forces to which [...] the organism had to become habituated […] In one sense, therefore, he might well have called his book *The Origin of Habits* rather than *The Origin of Species *(Huxley 1959, p. 496)

Although one should be wary of arresting statements such as Huxley's one about the title of Darwin's masterpiece *The Origin of Species*, the concept of habit does indeed play a pivotal role in Darwin's theory, recurring throughout his works from the early *Notebooks* (1836–1844) to the *Origin of Species* (first edition in 1859), the *Descent of Man* (1871), the *Expression of the Emotions in Man and Animals* (1872; see Browne 1985), up to his very last publication, *The Formation of Vegetable Mould through the Action of Worms, with Observations on their Habits* (1881), which appeared a few months before Darwin's death.

It was after he had returned to England from the *Beagle* voyage (1831–1836)^1^ that Charles Darwin took up the idea of species transmutation.^2^ Irresistibly drawn to the view, suggested by an impressive amount of evidence he had collected all over the world, that species change over time as they adapt to their changing environment, the young Darwin is in search of a mechanism to account for this transformation. His *Notebooks*, a fascinating collection of private notes, personal documents and short texts written between 1836 and 1844—among them, the *Red Notebook* (1836–1837), the *Transmutation Notebooks* (*B, C, D, E*, 1837–1839), the *Metaphysical Notebooks* (*M* and *N*, 1838–1840) the *Old and useless Notes about the moral sense and some metaphysical points* (1837–1840)—bear witness to the very first steps of this search. In *Notebook C*, the second *Transmutation Notebook*, we find a first vivid intuition of a possible mechanism: “habits give structure, therefore habits precede structure” (*C* 199),^3^ which means that it might be because of their habits that animals' anatomical structures change over time and, eventually, species evolve.

The mechanism seems to function as follows: when a species enters a new environment (or when the environment slowly alters), individuals are forced to adopt new habits to accommodate themselves to the new life conditions. These habits—recurrent patterns of behaviour performed, at least in their initial stages, with a certain degree of voluntariness—if practised over several generations (the environmental conditions remaining stable) gradually turn into more permanent behavioural features, which are involuntary, unconscious and transmissible to the offspring: instincts, or "*hereditary* habits" (see *Notebook N* 48: "Instinct is a modification of bodily structure"). Once embodied, these *hereditary habits* or instincts pave the way for the change of other anatomical structures, generating eventually a new species.

The young Darwin championed a genuinely materialistic view of the mind: “Why is thought being a secretion of brain more wonderful than gravity a property of matter?”, he asks rhetorically in *Notebook C* 166. In his view, every time we perform an action, the cognitive acts responsible for it leave a “mark” (a trace, a furrow) in the brain, which gradually—repetition after repetition—makes the related action smoother and also more and more involuntary and unconscious. In a word, the marks on the brain gradually turn the habitual action into an *instinct*, or *hereditary habit*:

When a muscle is moved very often, the motion becomes habitual and involuntary – when a thought is thought very often it becomes habitual and involuntary (Notebook M 46)

Resulting from the impact of recurrent (habitual) thoughts on the brain, these newly established neural furrows serve as the physical underpinnings of the corresponding animal instinct, now involuntary and innate. Darwin refers to instincts as a kind of “unconscious memory”, emerging from individual habits but—unlike individual habits *qua talis*—transmissible trans-generationally.^4^ Robert Richards, in his book *Darwin and the Emergence of Evolutionary Theories of Mind and Behavior*, recalls a pencilled annotation (probably written by Darwin in January 1840) to Johannes Müller's handbook *Elements of Physiology*, that sounds: “The inherited structure of brain must cause instincts: this structure might as well be bred as any other adapted structure” (Richards 1987, p. 95). In the young Darwin’s understanding, even the most spectacular animal instincts (such as egg-laying behaviour of common cuckoos or the slave-making instincts of certain ants) can be explained as sedimentation of habitual actions originally performed by the animal in a voluntary way, and which left their "mark", as a deep memory, on the animal’s brain: “Perhaps even the most complicated instinct might be analysed into steps, as species change” (*Old and Useless Notes* 37). *Notebooks C* and *D*, the second and third *Transmutation Notebooks*, are mainly devoted to examining the role of habits and instincts as agents of evolutionary change in animal behaviour and, consequently, in animal anatomy.

It should be emphasized that in Darwin's account *not* any habitual actions or habitual behaviours are per se entitled to turn into instincts: the sedimentation of habits into instincts is not a purely cumulative process. Indeed, two pre-conditions need to be fulfilled for a habit to be transformed into an instinct, generation after generation. First, the whole population, not just one individual or a couple of individuals, should take up the habit. In *Notebook C*, speculating about the hypothetical case of a jaguar that, because of the abundance of fish, gradually takes up the habit of swimming, Darwin writes (C 63): “Fish being excessively abundant and tempting the Jaguar to use its feet much in swimming, and every development giving greater vigour to the parent to tending to produce effect on offspring—but whole race of that species must take to that particular habit.—All structures either direct effect of habit, or hereditary and combined effect of habit,—perhaps in process of change.”

Second, the habit in question must be beneficial to the “race”: “It is probable that becomes instinctive which is repeated under many generations […] and only that which is beneficial to race will have recurred” (*Old and Useless Notes* 51), thus suggesting that useful habits, exactly because of their usefulness, are practised more frequently and therefore turned into instincts in succeeding generations. However, determining what is beneficial and to whom, at this very initial stage of Darwin's investigation, is not an easy task; this is why Darwin honestly admits, in the same passage: “until it can be shown what things easiest become instinctive, this part of argument fails, or rather is weak” (*Old and Useless Notes* 51).

Summing up from these quotations, it can be said that at the heart of Darwin's scientific investigations, as soon as he is back in England from his *Beagle* voyage, there is the idea that animal behaviour can be an agent of species change and that (at least some) habitual traits, features and abilities acquired over an individual's lifetime can be passed down to the offspring. Several questions, issues and doubts, however, remain to be addressed and full of hope but also aware of the difficulties of his approach to habits and instincts, Darwin writes in *Notebook C* (171):

Reflect much over my view of particular instinct being memory transmitted without consciousness, a most possible thing see man walking in sleep. an action becomes habitual is probably first stage, & an habitual action implies want of consciousness & will & therefore may be called instinctive. — But why do some actions become hereditary & instinctive & not others. We even see they must be done often to be habitual or of great importance to cause long memory. structure is only gained slowly. therefore it can only be those actions which many successive generations are impelled to do in same way. — The improvement of reason implies diversity & therefore would banish individual but general ones might yet be transmitted.

## Darwin, Lamarck and the Scottish transformism

It hardly needs mentioning that Darwin’s views on habits and instincts, at the time of his early speculations in the *Notebooks*, recall under many respects Jean Baptiste Lamarck's theory of habits. In *Notebook N* (91) Darwin admits: “Lamarck, habits becoming hereditary form the instincts of animals—almost identical with my theory, no facts, and mingled with much hypothesise”, thus suggesting that Lamarck's idea that animal habits gradually turn into instincts is not false per se, but it needs more accurate empirical grounding. Along the same line, in *Notebook C* 119 Darwin defines Lamarck “the Hutton of Geology”, “so bold” and “endowed with what may be called the prophetic spirit in science” but also, in the eyes of the young naturalist, an utterly speculative scientist, with very few clear facts and little evidence.

The influence of Lamarck’s transformism on the early stages of Charles Darwin’s evolutionary theory has been a fascinating and hotly debated topic among scholars, also because of the mixed and conflicting attitude of Darwin himself towards the French naturalist, documented in his private documents and writings (Burkhardt 1977, 2011, 2013; Corsi 1983; Jordanova 1984; Gissis and Jablonka 2011). In a famous letter to Joseph Dalton Hooker written on January 11 1844, Darwin writes: “I am almost convinced (quite contrary to opinion I started with) that species are not (it is like confessing a murder) immutable. Heaven forfend me from Lamarck nonsense of a «tendency to progression» «adaptations from the slow willing of animals» and c.,— but the conclusions I am led to are not widely different from his”.

An intriguing question is whether the first intellectual encounter between Darwin and Lamarck happened while the young naturalist was already about to leave for his *Beagle* voyage or even earlier. How and under the guidance of whom did the young Charles Darwin learn about French transformism? According to some recent research (Secord 1991; Jenkins 2015, 2016, 2019), it seems that the two years (1825–1827) that Charles Darwin spent as a medical student at the University of Edinburgh played in this respect an important role.^5^

While in Edinburgh Darwin read Lamarck, joined the Plinian Natural History Society, where radical ideas about the natural world, including Lamarck’s and Geoffroy Saint-Hilaire’s transformism, were discussed, and he was literally surrounded by enthusiastic supporters of transformist ideas, such as Robert Knox, Henry H. Cheek and Robert E. Grant, all active at the Medical School. Darwin and Grant, in particular, were relatively close friends and used to go together on invertebrate-collecting trips along the coast side of Edinburgh. We know from Darwin’s *Edinburgh Diary* that one day, during one of those trips, Grant “burst forth in admiration of Lamarck and his views on evolution”.

No doubt that among the Lamarckian topics discussed in Edinburgh there was Lamarck’s theory of habits and instincts and, more in general, his idea of the inheritance of acquired characteristics. For example, in a group of papers written by Henry Hulme Cheek (1807–1833), a medical student contemporary of Charles Darwin and member of the Royal Medical Society (which he joined on 3 November 1827, while Darwin on 17 November of the same year), we find interesting reformulations of Lamarck’s ideas on habits and transformism. In particular, there is a contribution, published by Cheek in the *Edinburgh Journal of Natural and Geographical Science* (edited by Cheek himself while a student), in which he discusses the relationship between living things and their conditions of existence. He writes: “All organized bodies possess the power of varying the development of the organs, by addition or subtraction of parts, as changes in the conditions of existence occur. It is easy to conceive that an organized body can assimilate elements in the form of a new organ, as new functions are required, when we recollect that it is constantly exercising a power of converting inorganic matter into the living emblem of its original form” (Jenkins 2015, p. 165). According to Jenkins (2015), these reflections by Cheek capitalize on Lamarck’s principle of use and disuse and on his theory of habits as mechanisms for the formation of new organs and, by means of this, of new species. In a series of recent papers and books, Jenkins has brilliantly reconstructed the Edinburgh “Lamarckian” atmosphere in the first half of the nineteenth century (also in the light of the traditionally very strict connections between France and Scotland, Edinburgh and Paris) and its possible impact on the young Charles Darwin during his two years as a student in Edinburgh (Jenkins 2015, 2016, 2019).

There is a point, as far as Lamarck’s and Darwin’s views on habits and instincts and the trans-generational inheritance of acquired habits are concerned, that we wish to stress. As we shall see in Sect. 7, contemporary epigeneticists supporting the idea that today's epigenetic theories shed new light on Darwin's early theories of habits and the idea of the transmission of acquired traits sometimes refer to epigenetics—particularly to the transgenerational inheritance of epigenetic markers—as to a form of "neo-Lamarckism" (Jablonka and Lamb 1995). However, Richard W. Burckhardt (2011) and Pietro Corsi (1983), among others, have convincingly shown that the idea that acquired, habitual characters can be transmitted over generations is not exclusively a "Lamarckian" theory, rather it was a relatively common view in the natural sciences between the eighteenth and the nineteenth century in France and Great Britain (Burkhardt 1977). Erasmus Darwin, for instance, was deeply convinced—of course without direct reference to Lamarck, since Erasmus’s main work *Zoonomia* appeared in 1794, fifteen years earlier than Lamarck’s *Philosophie Zoologique* (1809)—of the transmissibility of acquired habitual features from the parental generation to the offspring. Whereas, then, it is historically plausible that the first encounters with transformism, habits and the inheritance of acquired traits happened to Darwin through Lamarck in Edinburgh (and through his grandfather Erasmus), it is not true that the theory of the inheritance of acquired traits is per se a Lamarckian theory. This is why, throughout this paper, we will never refer to epigenetics as to a new or updated version of Lamarckism (Loison 2018).

## From habits to the *Origin*

The enthusiastic reception of the “Lamarckian” notion of habit and its extensive use in Darwin’s early writings were both radically overturned as soon as Darwin started to sketch out the idea of “natural selection” and its mechanisms of functioning. In the *Origin of species*, chapter VII of the first edition in 1859, Darwin writes that “It would be the most serious error to suppose that the greater number of instincts have been acquired by habit in one generation”. What lies behind this change of mind? First of all, it was not an easy transition.

According to Robert Richards, it took Charles Darwin no less than 20 years to set to one side his early theory of voluntary habits as the main engine of species transformation and to replace it with the principle of natural selection (Richards 1987, 1992, 2009). As known, Darwin reads for the first time Malthus’ *Principle of Population* in September 1838 and, inspired by this reading, starts to conceptualize the principle of natural selection and its role in the living world, which would lead him to the publication in 1859 of the first edition of his masterpiece, the *Origin of Species*. Evidence derived from Darwin's notebooks, from his letters and private documents suggests however that, along with natural selection, he also continued for many years (even after 1859) to give some credit to his older mechanism of inherited habits. Let us dwell for a moment on this point.

There is general agreement among scholars (see for instance Ospovat 1981; Manier 1978) that Darwin’s theory of evolution by means of natural selection did not come “out the blue”, rather it was bound up with and deeply influenced by the philosophical, theological and biological thought of his time. In that context, the notions of “habit” and “instinct” were commonly used by a vast array of thinkers (natural scientists, philosophers, natural theologians) to account for human and animal behaviour: John Mackintosh, Joseph Butler, William Kirby, William Spence, John Sebright, John Fleming, Henry Brougham (all of whom are mentioned in Darwin’s *Notebooks*), Edward Blyth, Erasmus Darwin himself, just to cite but a few examples,^6^ analyse, discuss, make use of the notions of habit and instinct in their works. To put it another way, habits and instincts were “established” concepts, which represented, for anyone interested in accounting for human and animal behaviour, a fundamental explanatory tool. It was quite obvious to the young Darwin to turn to them in his scientific investigations, to deepen his understanding of these notions while in Edinburgh and—more or less intentionally—to resist for a long time, even after the encounter with Malthus, any attempt to dismiss the habit principle and to replace it with a new one (Wright 2011).

Richards (1981) has argued that it was in particular the study of the works of some natural theologians of his time, namely the *Dissertations on Subjects of Science connected with Natural Theology* by Henry Brougham (1839) and the *Introduction to Entomology* by William Kirby and William Spence (1815–1825, in four volumes) that gradually undermined Darwin’s confidence in his early theory of instincts as hereditary (and originally voluntary) habits.

Brougham’s work urged Darwin to reflect more intensively on certain very sophisticated behavioural patterns, possessed by animals, which cannot be explained as deriving from previously acquired voluntary habits nor can be thought as being learnt by animals through a trial and error process. Let us consider, for instance, the marvellous instincts of the solitary wasp, which at a certain point in her life builds a nest to lay her eggs and supplies the eggs with grubs, although she will never see any “baby” wasp come out from the eggs (she normally dies before the hatching of the eggs) and although she has never tasted grubs before. How would it be possible, Darwin asks, to account for this behaviour as gradual sedimentation of habits? (Richards 1981; Morganti 2015, pp. 142–143). Even more challenging is the case of the spectacular instincts of the so-called neuter insects (asexual or sterile individuals, for example ant or bee workers), which cannot transmit their habits to offspring (since they are sterile) and have themselves parents endowed with quite different behavioural patterns (Ratnieks et al. 2010; Herb 2014). How did these neuter instincts originally arise and how can they be transmitted? Neuter insects are one of the examples discussed by Kirby and Spence in their *Introduction to Entomology*, a book that Darwin repeatedly praises, in his writings, for its acuity and clarity.

Apart from the precise individuation of the intellectual sources of Darwin’s rethinking of habits and instincts, it is a matter of fact, as mentioned, that chapter VII of the *Origin of Species* (first edition 1859) outlines the fundamentals of a “new theory of instincts”, different from what Darwin had suggested in the *Notebooks*. Darwin writes: “It would be the most serious error to suppose that the greater number of instincts have been acquired by habit in one generation, and then transmitted to succeeding generations. It can be clearly shown that the most wonderful instincts with which we are acquainted, namely, those of the hive-bee and of many ants, could not possibly have been […] acquired” through the experience of their utility, or with continued practice during successive generations. Rather, each instinct (and the related modifications in the species structure) should be understood as the result of the “accumulation of numerous, slight, and as we must call them accidental, variations, which are in any manner profitable, without exercise or habit having come into play. For no amount of exercise, or habit, or volition, in the utterly sterile members of a community could possibly affect the structure or instincts of the fertile members, which alone leave descendants” (*ibid*.).

In a few words, whereas in the *Notebooks* Darwin argues that certain voluntary habits are maintained because of their usefulness, gradually transformed into involuntary instincts and passed on to the offspring generation, in the *Origin of Species* he puts forward the idea that individuals endowed with advantageous modifications of a certain instinctive behaviour are gradually selected and their features trans-generationally transmitted. In Richard Lewontin’s terminology (1983), we witness here the transition from a *transformational* to a *variational* theory of species change. In transformational theories of change, species are seen as changing “because each individual organism within the species undergoes the same change” (let us recall what the young Darwin required as the first condition for species change, in the framework of his early theory of habits: that the whole “race”, not just one individual or a couple of individuals, takes up the beneficial habit); in variational theories “the individual members of the ensemble differ from each other in some properties and […] the system evolves by change in the proportions of the different types” (Lewontin 1983).

In the first edition of the *Origin of Species* (1859) the key principle of natural selection has largely absorbed the functions attributed to voluntary habits in the *Notebooks*. However, Darwin's downsizing of the role of voluntary habits in his masterpiece (and in the majority of his later works) should not imply that we ignore the significance attributed to this notion in his early writings. As we have mentioned, habits and instincts still have a role in the *Descent of Man* (1871), in the *Expression of the Emotions in Man and Animals* (1872; see Browne 1985) up to *The Formation of Vegetable Mould through the Action of Worms, with Observations on their Habits* (1881), which appeared a few months before Darwin's death. In this last book, Darwin discusses habits and behavioural patterns of earthworms, with a particular emphasis on their role in soil erosion, in the preservation of archaeological remains and the preparation of soil for plant growth. It has been argued that with this book Darwin anticipates the concept of what we call today “niche construction process theory” (Odling-Smee et al. 2003). But this is not the only contemporary theory that he seems to have anticipated with his (early and late) discussion on habits, as we will see in paragraph VII.

## Botanical habits: in support of the variational account

“It is rarely fully appreciated”, writes de Chadarevian (1996, p. 18), “that Darwin dedicated over twenty years to botanical investigation” and that, in his scientific activity, he was occupied with plants as much as with animals. Indeed, Darwin’s botanical work is considered by specialists still today of such great interest and relevance that “if he [Darwin] had produced no other works than these, posterity would have judged him a scientist of the first rank” (Ayres 2008, p. 11). Darwin’s publications on plants, from *The Various Contrivances by which Orchids Are Fertilised by Insects and the Good Effects of Intercrossing* (1862) to *The Movements and Habits of Climbing Plant* (first appeared in the ninth volume of the *Journal of the Linnean Society* in 1865 and then reprinted in an improved form in 1875 and in 1882), from the *Insectivorous Plants* (1875) to the *Effects of Cross and Self-fertilization in the Vegetable Kingdom* (1876) up to *The Power of Movement in Plants* (1880) testify to a deep interest in botanical projects that remained constant throughout his life.

Confronted with the widespread criticisms that started to be raised against him immediately after the publication of the first edition of the *Origin* (see Bellon 2009), it is precisely in botany that Darwin sought a much-needed weapon to counterattack. While already working on the book on orchids, in a letter from September 1861 to his publisher John Murray, Darwin explicitly admits: “I think this little volume will do good to the *Origin*, as […] it will, perhaps, serve [to] illustrate how natural History may be worked under the belief of the modification of Species” (Bellon 2009, p. 380). His botanical books then, although relevant in themselves and each endowed with its intrinsic value, are especially significant because of the examples and evidence they provided to support the arguments about natural selection that Darwin had already made in the first edition of the *Origin*.

Now, considering, on the one hand, how much “all-pervading” the concept of habit is in Darwin’s works (as we have shown above) and, on the other hand, how strictly related to his selection-orientated view is Darwin’s discussion of plants, we can expect—first—to find habits also applied to botanical matters and—second—to find the concept employed, in the botanical books, in the “variational” sense discussed in the final part of the preceding paragraph; even more so, since plants do not possess any kind of “voluntary” habits. This is exactly the case with Darwin’s botany: it is especially interesting because it provides a powerful illustration of what we have called, following Lewontin, Darwin’s variational theory of species change or—with specific reference to our subject in this paper—Darwin’s “new” variational theory of habits and instincts.

Let us briefly consider, in particular, the books *The Movements and Habits of Climbing Plant* (where the word “habit” stands out directly in the title) and *The Power of Movement in Plants*, which was published not long before Darwin’s death. In both works, “habit” is understood as a “spontaneous tendency”, a power (see, for instance, the frequent use that Darwin makes of the expressions “habit of climbing”, “habit of revolving”, “habit of circumnutating” etc.) which, through the gradual selection of advantageous individual variations, has become universal in the vegetable world because of its utility.

As known, Darwin in his botanical works rejects any distinction between the vegetable and the animal kingdom based upon the power of movement: “It has often been vaguely asserted”, he writes, “that plants are distinguished from animal by not having the power of movement” (Darwin 1882, p. 206). It should rather be said, Darwin contends, that every plant is able to move but each differs in the ways this power is displayed, according to how much of advantage to the plant it is. The fact that all plants have the power to move, i.e., the spontaneous tendency or habit to *circumnutate*, provides excellent evidence of the common ancestry theory, even in the case of very distantly related species.

“Circumnutation” is a technical term in Darwin’s botany, meaning basically “a revolving movement” (Darwin 1880, p. 1); the universal power to circumnutate, with which any plant is endowed, may be considered the ‘evolutionary precursor’ of all specific forms of movement (heliotropism, geotropism etc.) in the vegetable kingdom. As Darwin writes, “Circumnutation is of paramount importance in the life of every plant […]. As circumnutation is universally present, we can understand how it is that movement of the same kind have been developed in the most distinct members of the vegetable series”, for instance not only in plants with epigeal cotyledons (i.e., with embryonic leaves that grow above the ground) but also in vegetable species whose cotyledons are hypogeal. Darwin explains: “As this habit of the hypocotyl to arch itself appears to be universal, it is probably of very ancient origin. It is therefore not surprising that it should be inherited, at least to some extent, by plants having hypogean cotyledons, in which the hypocotyl is only slightly developed and never protrudes above the ground, and in which the arching is of course quite useless (Darwin 1880, p. 555). One of the main theses in Darwin’s botany sounds, therefore: “apparently every growing part of every plant is continually circumnutating, though often on a small scale” (Darwin 1880, p. 3).

Here Darwin’s variational assumption of the notion of habit is clear. There is no more room, after the *Origin* and with particular reference to the case of plants, for his early idea of voluntary tendencies that generation after generation may end up being fixed and become an instinct. On the contrary, plants’ “habit of circumnutating” is the result of the gradual selection over evolutionary time of spontaneous variations within plant populations, with the most advantageous becoming eventually universally widespread. In other words, since all plants need light to grow and since the habit of circumnutating enables plants to ascend to a height and thus to reach the light they need, all plants, generation after generation and thanks to the sieve of natural selection, end up with acquiring a habit or tendency to move, because of its utility (Darwin 1880, p. 266). This is true although, as said, many vegetable species display their power to move “only on a small scale”. Let us dwell for a moment on this point.

Towards the conclusion of his 1865 version of *The Movements and Habits of Climbing Plants*, (Darwin 1865, p. 114) and in the final 1882 version as well (Darwin 1882, p. 199), Darwin makes some interesting remarks: “during the endless fluctuations”, he writes, “in the conditions of life to which all organic beings have been exposed, it might have been expected that some climbing plants would have lost the habit of climbing. In the case of certain South African plants belonging to the great twining families, which in certain districts of their native country never twine, but reassume this habit when cultivated in England, we have a case in point. In the leaf-climbing *Clematis flammula,* and in the tendril-bearing Vine, we see no loss in the power of climbing, but only a remnant of that revolving-power which is indispensable to all climbers, and is so common, as well as advantageous, to most climbers”. This passage is important. Here Darwin suggests that, taken for granted the usefulness of the power to move and, therefore, its being universally favoured by natural selection, the external conditions of life can exert such a deep influence on plants that they may even lose, although temporarily, this important habit. In other words, he admits in this passage that the power of natural selection is not unlimited and that plants respond plastically to the fluctuations in the external conditions of life. The external conditions of life seem to be then, together with natural selection, a further factor in evolution. This view, here just briefly introduced by the great Victorian, plays an even more relevant role with reference to Darwin’s mature understanding of the notion of habit, as we shall see in the following paragraph.

## Back to the sterile workers

As shown in paragraph IV, it was plausibly the sterile workers problem, i.e. the question of the derivation of multiple forms of sterile workers within eusocial species, that in the 1840s slowly persuaded Darwin to set aside the idea of (voluntary) habits as the main agents of evolutionary change and to switch to a theory of habits and instincts based on natural selection processes. Chapter 7 of the *Origin* (Darwin 1859, p. 236) puts forward, as we have seen, a “new” theory of instincts based on natural selection.

There is no doubt that natural selection is, in Darwin’s understanding throughout the several editions of the *Origin*, the bedrock mechanism of adaptation. However, it took him not too long to realize that, although “the most important”, selection is not “*the exclusive* means of modification” (Darwin 1869, p. 6; see Ratnieks et al. 2010). Indeed, Darwin notoriously softens his appeal to natural selection across the six different editions of the *Origin*, showing to be more and more pluralist about the mechanisms of variation and adaptation.

With particular reference to the sterile workers problem, which, as argued in paragraph IV, plays a fundamental role in Darwin’s progressive reformulation of his early habit theory, the 1859 edition of the *Origin* (Darwin 1859, p. 236) speaks already rather clearly. While, on the one hand, Darwin presents here his new understanding of habits and instinct, he also admits, on the other hand, that there is “one special difficulty, which at first appeared to me insuperable, and actually fatal to my whole theory. I allude to the neuters or sterile females in insect-communities: for these neuters often differ widely in instinct and in structure from both the males and fertile females, and yet, from being sterile, they cannot propagate their kind”. To be sure, as demonstrated by Ratnieks et al. (2010), the “special difficulty” is not the origin of the sterile workers per se, rather how these traits—these “habits of sterility”—could be inherited.

Darwin goes on: “How the workers have been rendered sterile is a difficulty; but not much greater than that of any other striking modification of structure; for it can be shown that some insects and other articulate animals in a state of nature occasionally become sterile; and if such insects had been social, and it had been profitable to the community that a number should have been annually born capable of work, but incapable of procreation, I can see no very great difficulty in this being effected by natural selection. But I must pass over this preliminary difficulty […] *The great difficulty* lies in the working ants differing widely from both the males and the fertile females in structure, as in the shape of the thorax and in being destitute of wings and sometimes of eyes, and in instinct […] If a working ant or other neuter insect had been an animal in the ordinary state, I should have unhesitatingly assumed that all its characters had been slowly acquired through natural selection; namely, by an individual having been born with some slight profitable modification of structure, this being inherited by its offspring, which again varied and were again selected, and so onwards. But with the working ant we have an insect differing greatly from its parents, yet absolutely sterile; so that it could never have transmitted successively acquired modifications of structure or instinct to its progeny. It may well be asked *how is it possible to reconcile this case with the theory of natural selection*” (our emphasis). The “climax of the difficulty” is made explicit a few pages later (Darwin 1859, p. 238): “namely, the fact that the neuters of several ants differ, not only from the fertile females and males, but from each other, sometimes to an almost incredible degree, and are thus divided into two or even three castes. The castes, moreover, do not generally graduate into each other, but are perfectly well defined; being as distinct from each other, as are any two species of the same genus, or rather as any two genera of the same family”.

How is it possible to reconcile the “almost incredible” phenotypic plasticity of sterile workers, which we know today are all genetically identical, with the theory of natural selection? It is usually argued, in this respect, that Darwin anticipated what almost one hundred years later Hamilton (1964) would call “inclusive fitness theory”, since he writes in the *Origin* that we can solve the difficulty “when it is remembered that selection may be applied to the family, as well as to the individual” (Darwin 1859, p. 237). This is not, however, the line of argument that we want to follow here.

Let us go back to chapter 5 of the *Origin* but from edition 1869, not 1859. In chapter 5 (*Origin* 1869), dealing with the laws of variation, Darwin discusses the case of certain organisms belonging to the same species—such as our example here, i.e. sterile workers in eusocial species—that can adopt variable characters as a function of the “external conditions” they experienced. What Darwin calls “external conditions” corresponds to what is today called “environmental factors”, which are distinct from “genetic factors” and that Darwin distinguishes at that time as “the nature or the constitution of the organism” (Nicoglou 2014). Darwin writes: “In all cases there are two factors, the nature of the organism, which is much the most important of the two, and the nature of the conditions. The direct action of changed conditions leads to definite or indefinite results. In the latter case the organisation seems to become plastic and we have much fluctuating variability” (Darwin 1869, p. 166). As Nicoglou (2014) argues, even if he does not consider the direct action of ambient conditions as a factor that determines variation, Darwin nevertheless associates the organism’s plasticity with the “indeterminate” effects of changing external conditions on organisms. This means that, although he doesn’t refer to plasticity, Darwin is actually pointing out the concept of what we call today “plasticity”, associating it with “external conditions”. We know today that phenotypic plasticity is crucially linked to epigenetics and that epigenetic mechanisms are an important way in which the genome responds to environment factors (Duncan et al. 2014). Furthermore, we know (Herb 2014) that epigenetic mechanisms are the best explanation for the multiple forms of sterile workers: DNA methylation in honeybees and histone modifications in ants have been found to assist the formation of caste phenotypes during development and adulthood.

We can thus distinguish three different “movements” in Darwin’s treatment of the problem of the habits of sterility (and, together with this, of the habit problem in general). First, an attempt to explain the question through the gradual sedimentation of voluntary habits (in the *Notebooks*); second (for example in the *Origin of Species*, 1859, chap. 7), an explanation based on the “accumulation of numerous, slight, and as we must call them accidental, variations […] without exercise or habit having come into play”, which comes more than twenty years after the first, provisional formulation of the voluntary habit idea; third—for example, in *Origin* 1869—the introduction of a further factor (“external conditions in life”), which largely corresponds to what we call today plasticity and epigenetic mechanisms, to support and integrate the role of natural selection (chap. 5). In paragraph V we have already seen that the appeal to the “fluctuations in the external conditions of life” is crucial to explain the impressive variations among plants in the display of their (universal) habit of moving. It hardly needs to be said that plant epigenetics and the theory of epigenetic inheritance in plants is today an even more flourishing field of research than in animals (Henderson and Jacobsen 2007; Köhler and Springer 2017; Cruz and Becker 2020). Although this aspect would deserve more detailed exploration, we have decided in this paper to maintain our focus strictly on animals, i.e. on the role of habits in animal behaviour and, through this, in animal anatomy.

Summing up, by introducing a further factor of variation (“external conditions of life”) in his 1869 edition of the *Origin* in order to explain the habits of sterility of insect workers, Darwin seems to retrieve something of his early concept of habit (the idea of the malleability of organisms, the “indeterminate” effects of changing external conditions and the transmissibility of these variable, malleable characters), but within a framework which is now natural selection-orientated.

## Darwin’s early theory of habits and instincts under the lens of epigenetics

As mentioned in the introduction to this paper, renewed interest in Darwin's early transformational theories of habits and instincts has been recently observed in the field of epigenetics, which investigates the way in which the functioning and expression of genes is modified by environmental inputs and how these modifications are transmitted over generations (Bird 2007). Eva Jablonka, for instance, has written that through the lens of contemporary epigenetics we can appreciate how brilliant and ahead of his times was Darwin’s early idea of habits as drivers of the evolutionary process (Avital and Jablonka 2000, p. 30).

Today, indeed, there is growing empirical evidence that at least some of the habits that animals acquire during their lifetime can "leave a mark" on the genome, and, in certain cases, can be transmitted to the offspring (Matthews and Phillips 2010; Carey 2011; Qi et al. 2016). Epigenetic mechanisms have been shown to mediate the trans-generational inheritance of maternal care behaviour and stress reactivity in rats (Matthews and Phillips 2010). Habits of licking and grooming by rat mothers alter the DNA methylation of the glucocorticoid receptors in the pups (Matthews and Phillips 2010). These patterns of methylation affect gene expression and consequently brain development, with effects on the behaviour of the next generation of mother rats, which re-establishes the patterns of methylation in their pups. This is a case of trans-generational transmission of patterns of epigenetic modifications, resulting from repeated patterns of behaviour (“habitual behaviour”), without direct inheritance through the germline. Effects on the offspring of habits of maternal care, on the one hand, and of child abuse, on the other hand, probably mediated by the same epigenetic mechanisms, have been shown also in humans.

The transmission of acquired behavioural traits, however, can happen in mammals also through the germline (Sharma 2017). At present, the most remarkable evidence is provided by Brian Dias and Kerry Ressler (2014), who showed that when mice are taught to fear a specific odour, both their offspring and the next generation are born with a firm fear of the same smell due to the inheritance of a specific epigenetic modification occurring at the gene coding for the odorant receptor Olfr151.

As for human beings, an increasing number of studies have found direct and indirect evidence of the trans-generational transmission of epigenetic markers as a consequence of the exposure to a variety of conditions, such as traumatic experiences, poor nutritional habits, even tobacco smoke and mental and psychic disability (Pfeifer 2016). These experiences “of the fathers” literally *epigenetically* penetrate the body and are passed down to the offspring up to the second or third generation. One of the most remarkable examples is the (epigenetically mediated) transgenerational inheritance of the effects of malnutrition in the survivors of the Dutch Hunger Winter in 1944–1945, a period of terrible privations due to the German blockade of the Netherlands in the Second World War. Women that were pregnant during the famine (more specifically, women that suffered malnutrition in their *first months* of pregnancy) had children that, once grown up, showed a greater incidence of obesity, cardiovascular diseases, metabolic disorders. Even more interestingly, some of these health issues and problems seemed to affect also the survivors’ grandchildren. Studies examining DNA methylation patterns in the Dutch Hunger Winter survivors have found epigenetic changes in a set of genes involved in the regulation of metabolic activity (Heijmans et al. 2008; Veenendaal et al. 2013; Radford et al. 2014; Yehuda et al. 2016).^7^

There is an obvious difference between these pieces of empirical evidence about the transgenerational effects of epigenetically mediated “habits” and Darwin’s early transformational use of the notion: while the abovementioned studies focus on passive environmental exposures or forced choices such as famine and malnutrition, the young Darwin was more interested in voluntary, learned habitual actions and how they can be transmitted to the offspring, showing in this the influence of the Scottish philosophical milieu of his time (Wright 2011, 2017). Although not as massively as with cases of passive exposures, researchers have recently started, however, to suggest that epigenetic mechanisms might assure the “stabilization” of the effects also of active learning processes.

Broadly speaking, to acquire a “habit of learning” implies that we process repeatedly salient environmental cues and consolidate this salience to memory such that future encounters of these cues are met with appropriate behavioural outcomes. A relatively large set of data has assessed that epigenetic mechanisms play a pivotal role in shaping the functioning of neurons in mammals and, more specifically, the neural basis of memory formation, which is a fundamental component of learning processes. There has been an explicit proposal to read animal instincts as embodied *learned habits* (Robinson and Barron 2017), replacing the traditional “mutation first” model of the evolution of instincts (according to which a new innate behaviour can occur only when new mutations occur, i.e. variationally) with a “plasticity first” (or perhaps “transformational”) model of evolution, which holds that plasticity can precede, scaffold and facilitate evolutionary adaptations. We have indeed proofs that the genome responds dynamically to a range of behaviourally relevant stimuli, also with massive changes in the brain genes expression. But what could be the pros and what the cons of accepting such a “plasticity first” mechanism of evolution? One of the most frequent criticisms raised against epigenetic inheritance is that it is not stable enough to play a role in evolution, or, to put it differently, that it is not stable enough to give rise to new species. However, this instability should not be necessarily understood as a disadvantage. A “plasticity first” mechanism of evolution might be useful in many respects: for instance, an epigenetically-inherited behavioural trait can arise simultaneously in many individuals, as opposed to a single individual with a gene mutation; moreover, a transient epigenetically-modified phenotype can be relatively quick “reset”, when environmental conditions change, with individuals reverting to the original phenotype. As Stotz and Griffiths (2016) remark, to be reversible or less stable than genetic variation is not a downside per se: exactly because of their tentativeness, epigenetic mechanisms and epigenetically mediated behavioural patterns prove to be more sensitive to environmental fluctuation, more plastic, fluid, "creative", therefore more adaptive.

## Darwin, habits and the struggle for integration: a habit of doing science

Darwin’s early transformational use of the term “habit”, on the one hand, and contemporary research in epigenetics, on the other hand, have thus something in common, at least conceptually. Now, there have been recent attempts to “use” epigenetics as a kind of battering ram to counteract this or that fundamental assumption of Darwin’s theory of evolution and to suggest that, in the end, Darwin was wrong, acquired characters can indeed be transmitted, natural selection is not the undisputed ruler of evolution and genes are followers rather than drivers of evolutionary change.

In the last years, several scientists have suggested that an Extended Evolutionary Synthesis (EES) is required since the Modern Darwinian Synthesis (MDS) is not wide enough to accommodate new findings about epigenetic inheritance, plasticity, developmental constraints, or niche construction (Pigliucci 2005, 2010; Huneman 2019; Camacho 2020). Independently of the solution of this specific debate, we want to suggest here that Darwin’s flexible and pluralist approach to habits throughout his scientific activity, from his early *Notebooks* to the mature works, might have let him easily and profitably integrate within his theory even those mechanisms that go today under the label of epigenetic inheritance and, strictly connected to that, phenotypic plasticity. A case in point, in this respect, is Darwin’s treatment of the sterile workers problem (paragraph VI), i.e. how he came to terms with the problem of habits of sterility (and their transmission) in eusocial species.

We have seen, in paragraph VI, that Darwin did not hesitate to modify in a very substantial way his early understanding of the concept of habit as soon as new evidence and suggestions started to come from other naturalists (as described by Richards 1987) and from his own work on Malthus and natural selection (as shown in *Origin* 1859). Although with some initial resistance, due to specific appeal of the concept of habit, he eventually set aside his theory of voluntary habits and moved to a selection-based explanation in the first edition of the *Origin*.

Darwin was enough a responsible scientist, however, to recognize that there was nevertheless a “big difficulty” with this new variational and selection-orientated understanding. This is why, in the following editions of the *Origin* (such as the 1869 edition) and in other works of the 1860s and the 1870s, he gradually introduces other factors of evolution, together with adaptation and natural selection, to account for the splendid diversity we witness in the natural world and, more specifically, for the habits of sterility in insect workers. In so doing, he somehow went back conceptually to his early ideas on habits, although from a new, more mature perspective. The intriguing point we have highlighted in paragraph VII refers to the conceptual similarities that seem to emerge between some of these Darwin’s early ideas on habits and today’s research results in epigenetics.

Looked through the lens of “the further factors of evolution”—such as the effects of external conditions, the principle of use and disuse, the correlation of growth etc.—and although the laws of inheritance were unknown to Darwin and the same is, of course, true for epigenetics, our journey through Darwin’s discussion of habits leads, therefore, to the following conclusion: Darwin would have been keen to participate in the current debate about the role of epigeneticism (perhaps more prone to suggest that phenotypic plasticity is a result rather than a cause of variation in life making) and he would have inserted epigenetics in his original theory. By this way, he would have got the chance to understand better and into more detail one of the greatest challenges to his natural selection theory, i.e. sterile workers (Nicoglou 2014).

Darwin’s lesson about habits is, in this sense, not only conceptually but also and foremost methodologically relevant. How he dealt with this specific concept—“habit”—and with this specific problem—habits of sterility in eusocial species—throughout his scientific career, and how he struggled to come to terms with them and to integrate them within his natural selection theory reveals indeed, and relies on, a plastic *habit* of doing science. Without losing the overall coherence of his explanatory model, the Victorian tirelessly put forward ideas, retracted, retrieved and reformulated them according to evidence, always favouring the growth of science over dogmatic adherence to principles. It may perhaps even be said that he considered his own theory of habits (and, more in general, his own theory of evolution as a whole) itself a *habit*, i.e., something flexible which might be turned into a more fixed “instinct” but must not.

It is this pragmatic and plastic methodological approach to problems that we find most interesting in Darwin’s treatment of habits. As Lakatos (197, p. 175) wrote, mature scientific theories are those that struggle to grow, through positive and negative heuristics, and the “requirement of continuous growth” is the rational counterpart of “unity” or “beauty” in science. Capitalizing on these considerations, we argue that Darwin’s attitude to the habit problem might provide valuable methodological insights into the challenges that scientists face today in extending, expanding or revising the MDS, particularly in integrating epigenetic mechanisms into existing models of evolutionary change. His theory and practice of doing *flexible* science can work as a useful methodological paradigm, at least in the sense that, confronted with today’s epigenetics, Darwin would have surely concluded that there is nothing to be afraid of in it; rather, this is a stimulating research program to be developed.

We have tried to show in this paper, relying on Darwin’s texts, that it is not true, on the one hand, that the concepts of what we call today “epigenetics” and “phenotypic plasticity” have always been extraneous to Darwin’s thought (of course, under different names) and, on the other hand, that Darwin’s life-long “tormented” confrontation with habits and with the mechanisms of evolutionary change is not just a relevant chapter in the history of science but opens a fascinating window onto crucial matters of philosophy and methodology of contemporary scientific research.

## Footnotes

1 For 5 years, from 1831 to 1836, the young Charles Darwin circumnavigated the world aboard the HMS Beagle, a *Cherokee*-class 10-gun brig-sloop of the Royal Navy. “The voyage of the *Beagle* has been by far the most important event in my life and has determined my whole career”, writes Darwin in his *Autobiography* almost 40 years after he had disembarked from the *Beagle* (see http://darwin-online.org.uk/content/frameset?itemID=F1497&viewtype=text&pageseq=1).

2 But see, on Darwin’s engagement with transmutationist ideas already while aboard the *Beagle*, Brinkman (2009).

3 Throughout the present paper all references to Charles Darwin’s published works and manuscripts are taken from the online complete archive of his works, http://darwin-online.org.uk.

4 An important locus for the idea of instincts as an “unconscious memory” are Samuel Butler’s books *Evolution, Old and New* (1878) and *Unconscious memory* (1880). Butler, deeply influenced by evolutionary theories of his time, championed a neo-Lamarckian view of the evolution of the species.

5 On Lamarck’s reception in Great Britain, see Sloan Philipp, R., 1997, “Lamarck in Britain: Transforming Lamarck’s Transformism”, in *Jean-Baptiste Lamarck: 1744–1829*, Goulven Laurent (ed.), Paris: Éditions du CTHS, 667–687.

6 Needless to say, David Hume’s in-depth and decisive treatment of the notion of “habit” lies behind the vast majority of these thinkers’ views and ideas.

7 In this sense, traumas, malnutrition, bad habits, and poor health conditions might be considered, in certain cases at least, “sins of the fathers that are to be laid upon the children”, with obvious ethical and social implications (“intergenerational responsibility”), and a rapidly growing field of research in the social sciences that looks at epigenetics as a challenge and a new stimulating area of interest on the boundary between nature and nurture (see Richardson 2017; Lock 2012; Tolwinski 2013).
